# Towards reference values of pericoronary adipose tissue attenuation: impact of coronary artery and tube voltage in coronary computed tomography angiography

**DOI:** 10.1007/s00330-020-07069-0

**Published:** 2020-07-22

**Authors:** Runlei Ma, Daan Ties, Marly van Assen, Gert Jan Pelgrim, Grigory Sidorenkov, Peter M. A. van Ooijen, Pim van der Harst, Randy van Dijk, Rozemarijn Vliegenthart

**Affiliations:** 1 Department of Radiology, University of Groningen, University Medical Center Groningen, Hanzeplein 1, 9713 GZ Groningen, the Netherlands; 2 Department of Radiology, Affiliated Hospital of Nanjing University of Chinese Medicine, Nanjing, China; 3 Department of Cardiology, University of Groningen, University Medical Center Groningen, Groningen, the Netherlands; 4 Department of Epidemiology, University of Groningen, University Medical Center Groningen, Groningen, the Netherlands; 5 Department of Radiation Oncology, University of Groningen, University Medical Center Groningen, Groningen, the Netherlands; 6 Data Science Center in Health, University of Groningen, University Medical Center Groningen, Groningen, the Netherlands

**Keywords:** Computed tomography angiography, Adipose tissue, Coronary vessels/diagnostic imaging, Reproducibility of results, Atherosclerosis

## Abstract

**Objectives:**

To determine normal pericoronary adipose tissue mean attenuation (PCAT_MA_) values for left the anterior descending (LAD), left circumflex (LCX), and right coronary artery (RCA) in patients without plaques on coronary CT angiography (cCTA), taking into account tube voltage influence.

**Methods:**

This retrospective study included 192 patients (76 (39.6%) men; median age 49 years (range, 19–79)) who underwent cCTA with third-generation dual-source CT for the suspicion of CAD between 2015 and 2017. We selected patients without plaque on cCTA. PCAT_MA_ was measured semi-automatically on cCTA images in the proximal segment of the three main coronary arteries with 10 mm length. Paired *t*-testing was used to compare PCAT_MA_ between combinations of two coronary arteries within each patient, and one-way ANOVA testing was used to compare PCAT_MA_ in different kV groups.

**Results:**

The overall mean ± standard deviation (SD) PCAT_MA_ was − 90.3 ± 11.1 HU. PCAT_MA_ in men was higher than that in women: − 88.5 ± 10.5 HU versus − 91.5 ± 11.3 HU (*p* = 0.001). PCAT_MA_ of LAD, LCX, and RCA was − 92.4 ± 11.6 HU, − 88.4 ± 9.9 HU, and − 90.2 ± 11.4 HU, respectively. Pairwise comparison of the arteries showed significant difference in PCAT_MA_: LAD and LCX (*p* < 0.001), LAD and RCA (*p* = 0.009), LCX and RCA (*p* = 0.033). PCAT_MA_ of the 70 kV, 80 kV, 90 kV, 100 kV, and 120 kV groups was − 95.6 ± 9.6 HU, − 90.2 ± 11.5 HU, − 87.3 ± 9.9 HU, − 82.7 ± 6.2 HU, and − 79.3 ± 6.8 HU, respectively (*p* < 0.001).

**Conclusions:**

In patients without plaque on cCTA, PCAT_MA_ varied by tube voltage, with minor differences in PCAT_MA_ between coronary arteries (LAD, LCX, RCA). PCAT_MA_ values need to be interpreted taking into account tube voltage setting.

**Key Points:**

*• In patients without plaque on cCTA, PCAT*_*MA*_ *differs slightly by coronary artery (LAD, LCX, RCA).*

*• Tube voltage of cCTA affects PCAT*_*MA*_ *measurement, with mean PCAT*_*MA*_ *increasing linearly with increasing kV.*

*• For longitudinal cCTA analysis of PCAT*_*MA*_ *, the use of equal kV setting is strongly recommended.*

**Electronic supplementary material:**

The online version of this article (10.1007/s00330-020-07069-0) contains supplementary material, which is available to authorized users.

## Introduction

Coronary artery disease (CAD) is caused by atherosclerosis of the coronary arteries. Prior studies showed that coronary inflammation plays an essential role in the development and progression of atherosclerotic plaque [1–3]. An observational study demonstrated that invasively determined inflammatory changes of the coronary wall are present in early stages of CAD [4]. In the CANTOS trial, anti-inflammatory therapy reduced cardiovascular events, independent of lipid-lowering therapies [5, 6]. Efforts have been made to find a reliable non-invasive imaging parameter to detect coronary inflammation, focusing on adipose tissue [7–10]. The amount of epicardial adipose tissue (EAT) has been quantified [11–13], not only based on coronary computed tomography angiography (cCTA) but also based on coronary calcium scans or non-gated chest CTs [14, 15]. More recently, attention was focused on pericoronary adipose tissue (PCAT). Although PCAT is part of EAT, morphological and functional characteristics of PCAT are different from those of EAT. PCAT is directly affected by coronary inflammation, causing compositional changes of PCAT, while EAT is mainly affected by systemic conditions such as obesity [16]. A clinical pathology review suggested PCAT to be an independent risk factor for cardiovascular disease [17]. Antonopoulos et al indirectly evaluated coronary inflammation on cCTA around the RCA by measuring the fat attenuation index, equivalent to PCAT mean attenuation (PCAT_MA_) [16]. They found a correlation between cCTA-derived PCAT_MA_ and adipocyte size or PCAT lipid volume in ex vivo PCAT histology. Additionally, PCAT and the coronary wall had a bidirectional communication, where inflammatory processes in the coronary vessel wall influenced PCAT composition via a paracrine pathway [16, 18]. In turn, PCAT influenced the coronary wall by secreted bioactive inflammation molecules [19]. In the presence of increased inflammation, higher CT attenuation of PCAT is expected [16].

Thus, PCAT could potentially be used as a non-invasive proxy to assess coronary inflammation based on routine cCTA imaging, and could offer valuable information for early diagnosis, treatment, and prevention of CAD. Several studies explored the diagnostic value of PCAT_MA_ in patients with plaques [20–24]. However, studies including patients without plaque so far mainly focused on the healthy RCA in small cohorts of patients. Further standardization and validation, as well as reference PCAT_MA_ values in all three main coronary arteries without plaque, are needed before generalized clinical implementation can be considered. Reference values of PCAT_MA_ for healthy patients are necessary for the application in diseased patients because based on the healthy reference values clinicians may in the future be able to classify the coronary arteries into healthy or vulnerable vessels, even before the presence of plaque. Additionally, clinical cCTA scans are acquired at different tube voltages depending on patient characteristics and CT systems. Differences in tube voltage affect Hounsfield Unit (HU) values measured in different tissues. However, so far, no study has actually studied the magnitude of the effect of the tube voltage on PCAT. Potential future cutoff values in PCAT need to be seen in perspective of difference by tube voltage, and may need adjustment by kV setting.

The objectives of this study were to explore PCAT_MA_ reference values of three main coronary arteries in patients without plaque on cCTA, and to determine the influence of cCTA tube voltages and vessel analyzed on PCAT_MA_ measurement.

## Materials and methods

### Study population

This retrospective, single-center observational study was performed at the University Medical Center Groningen, Groningen, The Netherlands. The study was compliant with the Declaration of Helsinki. The study protocol was approved by the institutional ethical review board, and informed consent was waived. Patients were eligible if they were suspected of coronary artery disease and underwent routine cCTA between January 2015 and November 2017. The cohort list was randomly screened for patients meeting the inclusion criteria until the required sample size was reached; see sample size calculations in the statistical paragraph. Inclusion criteria were (1) calcium score of 0 and (2) no coronary plaque on cCTA. Exclusion criteria were (1) objection to the use of data for scientific research; (2) poor cCTA image quality; and (3) patients with anomalous coronary artery origin from the aorta sinus that leads to inaccurate measurements. A radiologist with 10 years of experience re-evaluated all calcium scoring and cCTA scans. In case of doubt, a radiologist with 14 years of experience performed a second reading.

### cCTA scan and post-processing protocol

Third-generation dual-source CT was used (Somatom Force; Siemens Healthineers). A non-enhanced electrocardiography (ECG)-triggered CT acquisition was performed to obtain the calcium score. cCTA was performed according to standard clinical protocol. Patients received sublingual nitroglycerin unless contra-indicated. In case of high heart rate (> 70–73 beats/min), patients received an intravenous beta-blocker. cCTA was acquired in high-pitch mode in case of regular heart rate < 70 beats/min, in sequential mode in diastolic phase if heart rate was > 70 beats/min, and in sequential mode with broad ECG interval in case of arrhythmias. Tube voltage ranged from 70 to 120 kV, depending on patient size, as suggested by CarekV (kV optimization assistance). Contrast bolus timing was determined after a test bolus. Iomeprol (Iomeron 350; Bracco Altana Pharma) was injected with dose and flow rate depending on patient characteristics and scan mode. A dual bolus technique was used followed by a saline flush. cCTA images were reconstructed with a slice thickness of 0.6 mm. Post-processing and analysis of cCTA images were performed using dedicated software (Aquarius iNtuition, TeraRecon, Version 4.4.13).

### PCAT_MA_ measurement

**Fig. 1:**
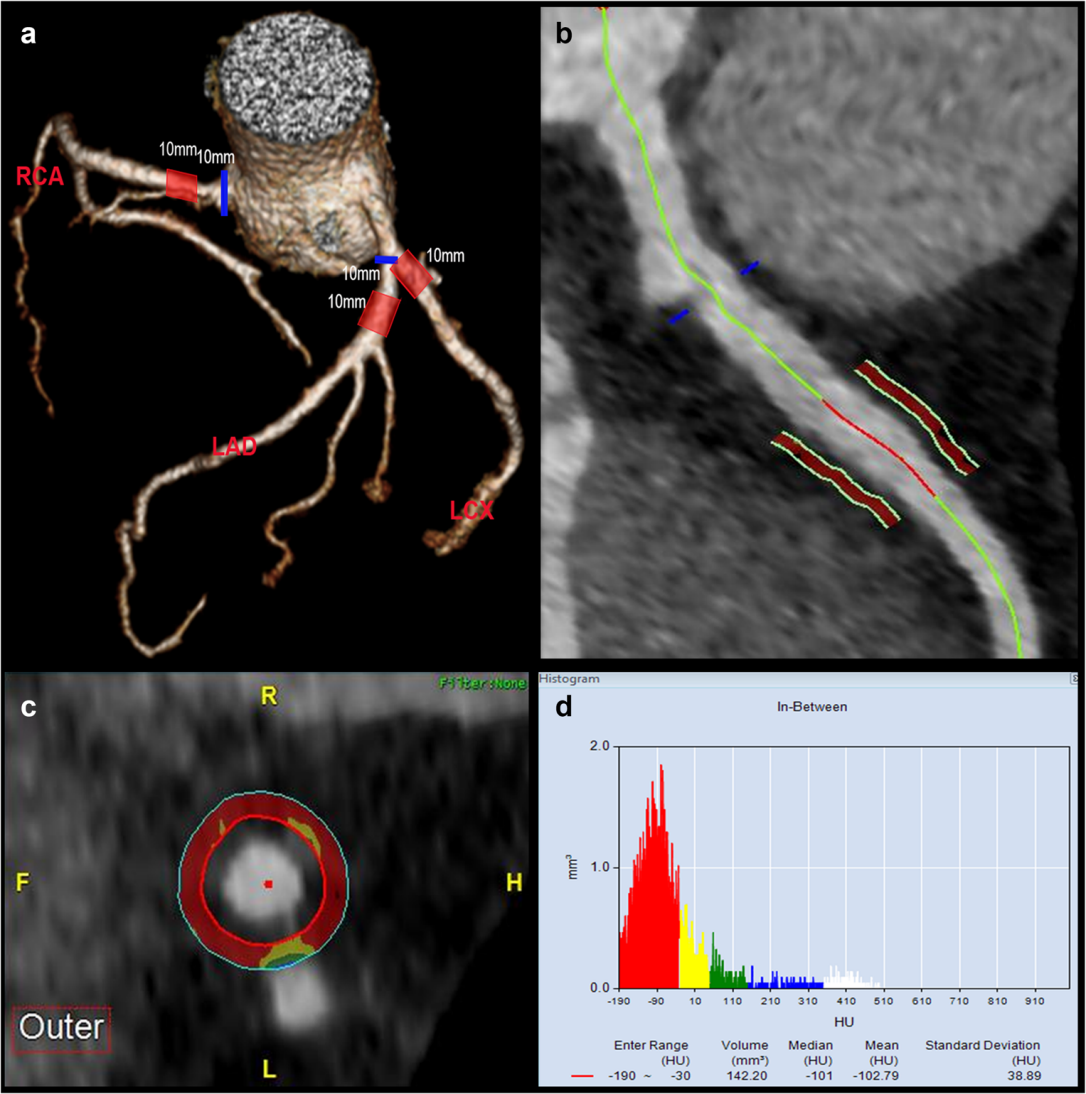
Measurement steps of PCAT_MA_ (cCTA at 70 kV in a 56-year-old male patient). (**a**) Measurement ranges (red rectangles) are marked on the VR image, the 10-mm reference line is the blue line. (**b**) A gap of 1 mm is determined around the border of the coronary lumen. (**c**) CT density is measured for a concentric ring from 1 to 2 mm around the coronary lumen (1 mm thickness). (**d**) The software automatically calculates the mean CT attenuation and volume for voxels within the target threshold of − 190 to – 30 HU. PCAT_MA_ is pericoronary adipose tissue mean attenuation; LAD is left anterior descending coronary artery; LCX is left circumflex coronary artery; RCA is right coronary artery; VR is volume rendering; cCTA is coronary computed tomography angiography

PCAT_MA_ was measured in the main coronary arteries (LAD, LCX, and RCA). PCAT_MA_ measurements were based on the conceptual framework as proposed by Antonopoulos et al [16]. The workstation automatically reconstructed three-dimensional volume-rendered and curved multi-planar reformat images, which were manually corrected by the radiologist in case of identification errors. The following steps were performed (Fig. 1): (a) Start and end points of the PCAT_MA_ measurement were selected. For LAD and RCA, the start point was 10 mm distally from the origin to avoid overlap with the LCX measurement and influence of the aortic wall, respectively. For LCX, the vessel origin was selected as the start point. Because of LCX anatomy, there is limited adipose tissue around the LCX after 10 mm. We adjusted the measurement length to 10 mm for all coronary arteries in order to minimize interference of side branch intersections, which costs less time than the 40 mm in the original study [16]. (b) A 1-mm gap was left around the artery lumen and the measurement circle in order to prevent blooming artifacts from high contrast concentration. (c) The mean HU value of adipose tissue was measured in a concentric circle from 1 to 2 mm around the coronary lumen (1 mm thickness). Compared with prior studies, we reduced measurement width from the average vessel diameter of about 3 to 1 mm in order to avoid interference from the myocardium and veins. (d) The software automatically calculates the mean CT attenuation and volume for voxels within the target threshold of − 190 to – 30 HU [16].

### EAT measurements

The heart was manually segmented. Within this segmentation, an HU range from − 190 to − 30 was set to select relevant tissue. The volume value of the EAT was automatically obtained by the software.

### PCAT measurement methodology variation

In order to evaluate whether measurement length influences PCAT_MA_ measurements, PCAT_MA_ was measured with 40 mm and 10 mm lengths in 60 randomly selected cCTA scans. In twenty randomly selected cCTAs, intra- and inter-observer agreements were determined. For intra-observer agreement, PCAT_MA_ was measured again by the same reader after at least 4 weeks to avoid image recognition. For inter-observer agreement, a second independent reader measured PCAT_MA_ after sufficient training.

### Statistical methods

The sample size was calculated using paired sample *t*-testing with GPOWER software (Faul, Erdfelder, Lang, & Buchner, version 3.1.9.2). For sample size calculation, we used results from two prior PCAT_MA_ studies [16, 25], with the following parameters: mean and SD of PCAT_MA_ (− 75.1 ± 8.6 HU and − 77.0 ± 8.5 HU); correlation between groups 0.5. The effect size was calculated to be 0.2222108. With *α* = 0.05, power = 0.8, and two-tailed analysis, the needed sample size was 161. We added 20%, yielding a total sample size of 192, to decrease type I and type II error ratios.

Normality testing for continuous variables was performed with the Shapiro-Wilk test. Continuous variables were represented as mean ± SD. Categorical variables were recorded as numbers (*n*) and frequencies (%). Associations of age, sex, and BMI with PCAT_MA_ were tested using multivariable regression analysis. PCAT_MA_ values by sex were compared using unpaired sample *t*-testing. PCAT_MA_ comparisons between combinations of two coronary arteries were made using paired sample *t*-testing; values between three coronary arteries were compared using repeated ANOVA testing. In order to determine the effects of tube voltage on PCAT_MA_, patients were grouped according to tube voltage as follows: 70, 80, 90, 100, 120 kV. For PCAT_MA_ and EAT volume comparison of multiple kV groups, one-way ANOVA testing was used. Post hoc pairwise comparisons of PCAT_MA_ were performed between each two kV groups. *p* values < 0.05 were considered statistically significant. For multi-paired *t*-testing a Bonferroni correction was applied, adjusting the *p* value accordingly. SPSS (SPSS, version 25; IBM) was used for statistical analysis.

## Results

### Study population characteristics

**Fig. 2:**
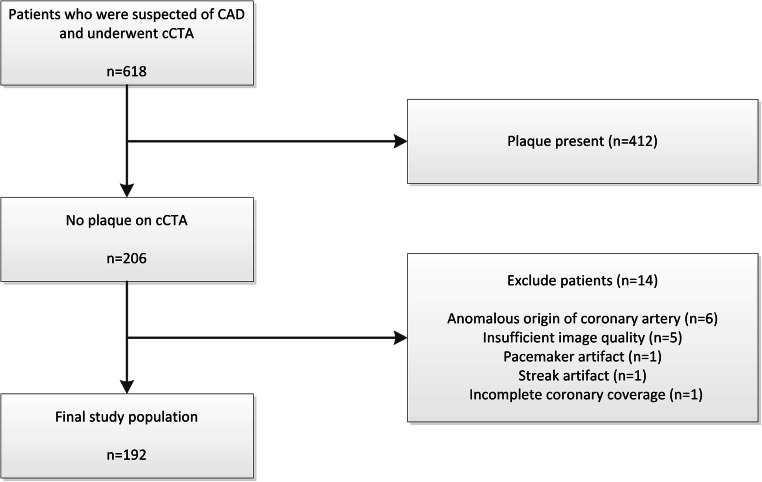
Flowchart of patient inclusion. CAD is coronary artery disease; cCTA is coronary computed tomography angiography

In total, 206 patients without CAD on cCTA images were selected for analysis. Fourteen patients were excluded for various reasons: anomalous origin of coronary artery (*n* = 6), insufficient image quality (*n* = 5), incomplete coronary image coverage (*n* = 1), pacemaker artifact (*n* = 1), and streak artifact (*n* = 1) (Fig. 2). The final study population consisted of 192 patients (76 [39.6%] men; mean age 50.5 years [range, 19–79 years]) and 576 coronary arteries. Overall, 72 patients (37.5%) underwent cCTA at 70 kV, 53 (27.6%) at 80 kV, 39 (20.3%) at 90 kV, and 28 (14.6%) at 100 to 120 kV (Table 1).

**Table 1:** Patients’ baseline characteristics. Body mass index information was available for 108 patients. *kV* is tube voltage, *SD* is standard deviation, *cCTA* is coronary computed tomography angiography

Variables	Overall (*n* = 192)
Age, years, mean ± SD	50.5 ± 11.5
Men, *n* (%)	76 (39.6%)
Body mass index, mean ± SD	26.4 ± 5.0
Risk factor, *n* (%)	
Diabetes mellitus	13 (6.8%)
Hypertension	70 (36.5%)
Hyperlipidemia	34 (17.7%)
Former smoker	38 (19.8%)
Current smoker	37 (19.3%)
Family history of coronary artery disease	68 (35.4%)
Indication for cCTA, *n* (%)	
Typical angina	15 (7.8%)
Atypical angina	100 (52.1%)
Non-anginal chest pain	14 (7.3%)
Dyspnea/dyspnea’ effort	12 (6.3%)
Other	51(26.6%)
Tube voltage, *n* (%)	
70 kV	72 (37.5%)
80 kV	53 (27.6%)
90 kV	39 (20.3%)
100–120 kV	28 (14.6%)

### PCAT_MA_ of healthy coronary arteries on cCTA

**Fig. 3:**
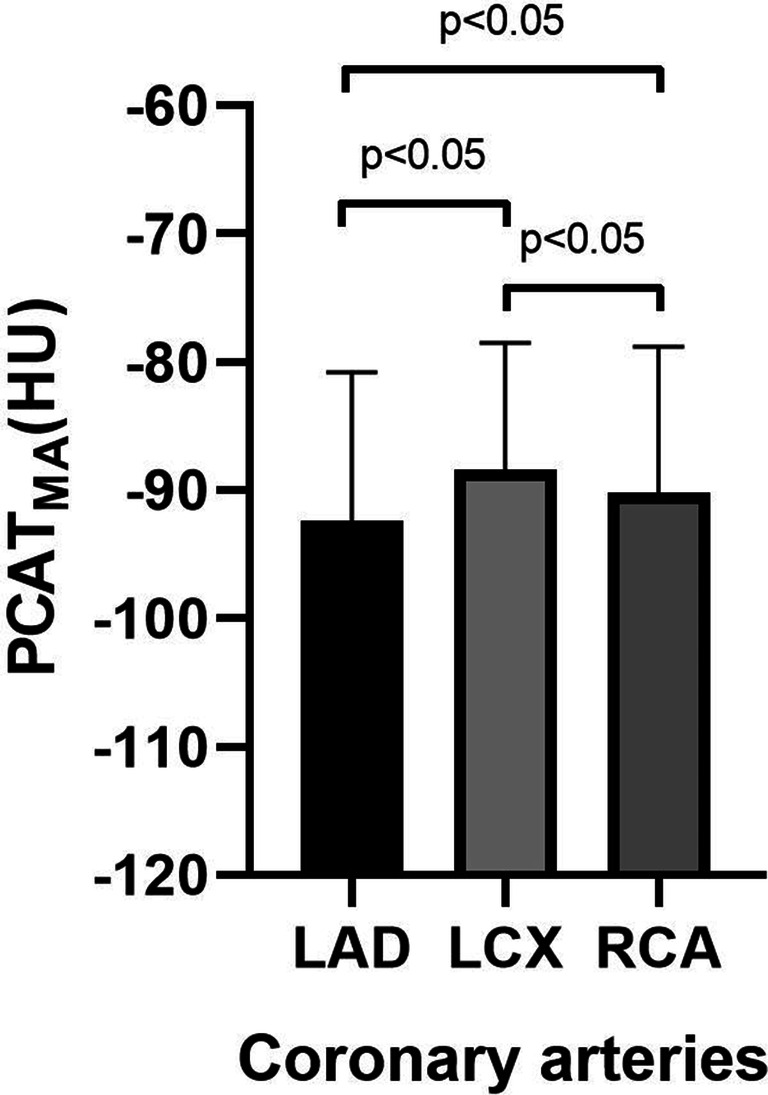
PCAT_MA_ values for the main coronary arteries. PCAT_MA_ is pericoronary adipose tissue mean attenuation; LAD is left anterior descending coronary artery; LCX is left circumflex coronary artery; RCA is right coronary artery

Overall mean PCAT_MA_ value was − 90.3 ± 11.1 HU. Mean PCAT_MA_ of men and women was − 88.5 ± 10.5 HU and − 91.5 ± 11.3 HU (*p* = 0.001), respectively. In multivariable linear regression analysis, kV, age, and gender were significantly associated with PCAT_MA_ (*p* < 0.05) while BMI was not (*p* = 0.235). Mean PCAT_MA_ of LAD, LCX, and RCA was − 92.4 ± 11.6 HU, − 88.4 ± 9.9 HU, and − 90.2 ± 11.4 HU, respectively (*p* < 0.001). There were significant differences between all combinations of coronary arteries: PCAT_MA-LAD_ and PCAT_MA-LCX_ (*p* < 0.001), PCAT_MA-LAD_ and PCAT_MA-RCA_ (*p* = 0.009), PCAT_MA-LCX_ and PCAT_MA-RCA_ (*p* = 0.033) (Fig. 3).

### Influence of tube voltage on PCAT_MA_

**Fig. 4:**
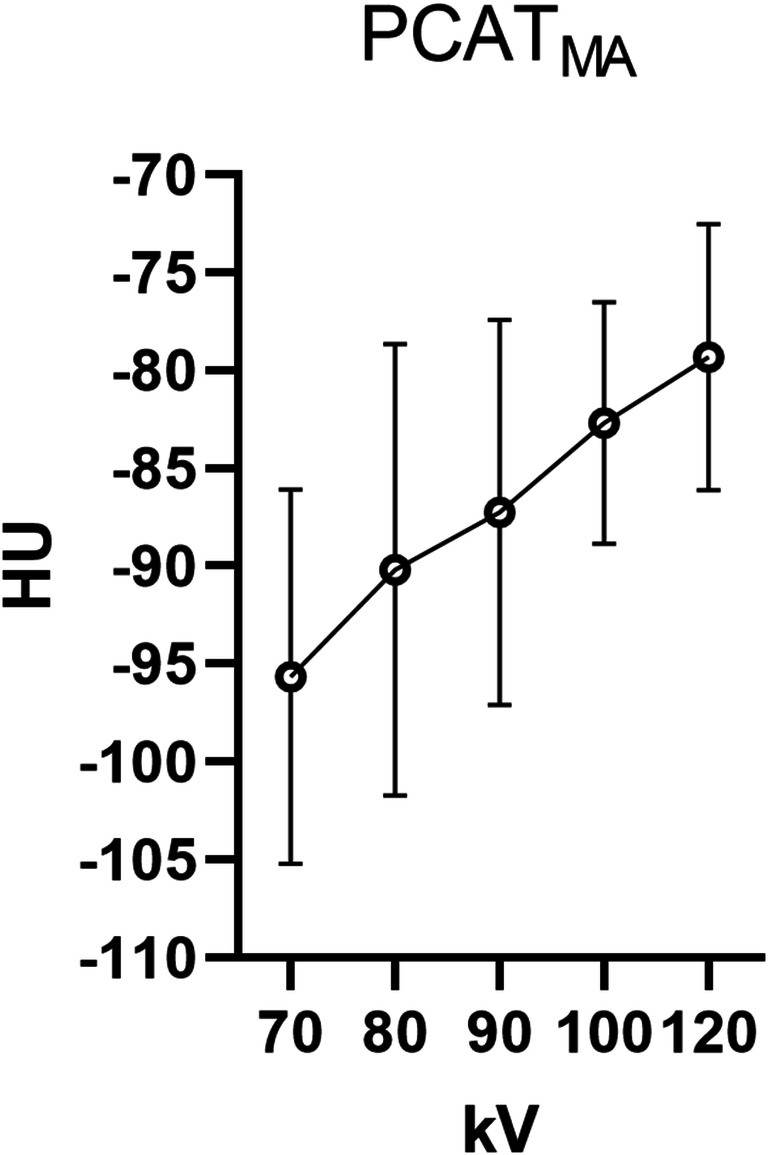
PCAT_MA_ values in patient groups based on cCTA tube voltage setting. PCAT_MA_ is pericoronary adipose tissue mean attenuation; kV is kilovoltage; HU is Hounsfield Units; cCTA is coronary computed tomography angiography

Mean PCAT_MA_ showed a positive linear association with tube voltage (Fig. 4). Mean (SD) PCAT_MA_ of the 70 kV, 80 kV, 90 kV, 100 kV, and 120 kV groups was − 95.6 ± 9.6 HU, − 90.2 ± 11.5 HU, − 87.3 ± 9.9 HU, − 82.7 ± 6.2 HU, and − 79.3 ± 6.8 HU, respectively (*p* < 0.001). Post hoc pairwise comparisons of the kV groups demonstrated significant differences between each two groups except for the 80 kV and 90 kV (*p* = 0.222), and 100 kV and 120 kV groups (*p* = 0.267).

### Tube voltage and EAT volume

**Fig. 5:**
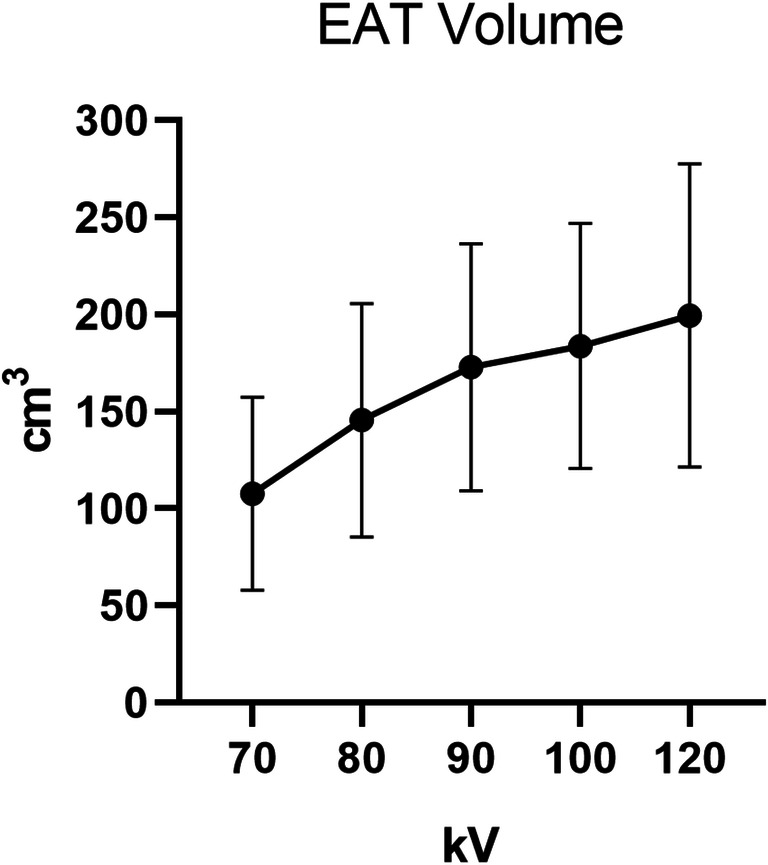
EAT volume in patient groups based on cCTA tube voltage setting. EAT is epicardial adipose tissue; kV is kilovoltage; CM is centimeter; cCTA is coronary computed tomography angiography

Mean EAT volume showed a positive linear association with tube voltage (Fig. 5). Mean (SD) EAT volume of the 70 kV, 80 kV, 90 kV, 100 kV, and 120 kV groups was 107.6 ± 49.7 cm^3^, 145.5 ± 60.1 cm^3^, 172.8 ± 63.6 cm^3^, 183.7 ± 63.2 cm^3^, and 199.5 ± 78.1 cm^3^, respectively (*p* < 0.001).

### PCAT measurement methodology variation

PCAT_MA_ of LAD, LCX, and RCA for 40-mm measurement length was − 95.6 ± 9.7 HU, − 88.7 ± 10.0 HU, and − 92.9 ± 8.3 HU, respectively, compared with − 94.5 ± 11.0 HU, − 90.0 ± 8.7 HU, and − 91.6 ± 9.7 HU for 10-mm measurement length (*p* = 0.124, 0.118, 0.116, respectively) (Supplementary Material Table S1 and Fig. S1). There was excellent correlation within and between readers for repeated PCAT_MA_ measurements (0.974–0.982), with minimal bias but with some variability between readings (upper and lower limits of agreement 3.9 HU and − 3.3 HU within-reader, 4.6 HU, and − 4.1 HU between-readers) (Supplementary Material Table S2 and Fig. S2).

## Discussion

In this study, we investigated the PCAT_MA_ values of healthy coronary arteries and the influence of tube voltage. Our main results showed that the tube voltage of cCTA significantly influenced PCAT_MA_ values in patients without CAD, and that PCAT_MA_ differed slightly between the LAD, LCX, and RCA.

Although the presence of obstructive disease on cCTA is associated with worse outcomes, many myocardial infarctions originate from coronary segments without prior obstructive disease. Thus, the focus has shifted to the identification of segments at future risk of developing potentially vulnerable plaque [26]. Studies on PCAT_MA_ in diseased populations showed significant differences between diseased and non-diseased coronary arteries, and between flow-limiting and non-flow limiting stenosis [24]. Although results were statistically significant, these studies show, similar to the current study, limited absolute differences in PCAT values (± 5 HU). However, these studies show an increased accuracy for the prediction of hemodynamic significance of a lesion, especially in combination with other factors such as stenosis diameter. Further understanding of the PCAT parameter and the influence of scan protocol settings on this biomarker and its variability can help to determine limits of reliability around PCAT_MA_ values when comparing patients.

One of the main results of this study is that the use of different kV levels has considerable impact on PCAT_MA_ values. In clinical practice, cCTA acquisitions are acquired with varying kV levels. Higher kV voltages will inherently lead to higher PCAT_MA_ and EAT attenuation, unrelated to a pathophysiological process. Prior studies investigating the use of PCAT_MA_ used cCTA images obtained at 100 kV and/or 120 kV [16, 20, 21, 25]. However, lower kV acquisitions are becoming increasingly popular in order to reduce radiation and contrast medium volume. Recent results from the PROTECTION VI Study showed that low kV settings for cCTA (< 100 kV) are already applied in 14% of patients and this is only expected to increase [27, 28]. Our results showed significant differences between all kV levels except between 80 and 90 kV, and between 100 and 120 kV. The lack of difference in PCAT_MA_ between 100 and 120 kV is also reflected by the similar PCAT_MA_ results from previous studies investigating only those two levels. The differences in PCAT_MA_ between the other kV levels indicate that a kV-specific PCAT_MA_ cutoff should be used to discriminate healthy from diseased patients and perform accurate risk assessment. Determination of this cutoff fell outside the scope of this research. The current study provides reference values of PCAT_MA_ by kV setting in normal coronary arteries; future studies should investigate the PCAT_MA_ by kV in coronary arteries with plaque and/or stenosis, and evaluate the optimal cutoff values. To construct a kV correction factor, ideally the same patients would have to undergo repeated cCTA at different kV levels. The results also showed a positive relation between kV and EAT volume. This is likely mostly due to the fact that patients with higher BMI (and more intrathoracic fat) were usually scanned with higher kV to get better image quality. In view of the influence of kV on PCAT_MA_ analysis, it is recommended that for the longitudinal follow-up and comparison of cCTA-based PCAT_MA_ values, the cCTA is performed at equal tube voltage setting.

The majority of PCAT_MA_ studies about relationship with CAD focused on analysis of the RCA alone, while the LAD and LCX could provide additional information and increase the accuracy of outcome prediction [16, 21]. Our study focused on PCAT_MA_ measurements in all three coronary arteries, showing that PCAT_MA_ was slightly but significantly different between the LAD, LCX, and RCA. This difference could be caused by differences in anatomy and surrounding tissues, indicating that PCAT_MA_ values and corresponding cutoff values based on RCA measurements cannot be directly transferred to the other coronary arteries. Our study results showed that LAD had slightly but significantly lower PCAT_MA_ compared with the RCA and LCX. As is known in literature, atherosclerosis development also differs between the coronary arteries. The LAD is subject to atherosclerosis more often and at an earlier stage in comparison with RCA and LCX [29–31]. The fact that LAD had a lower PCAT_MA_ is an important hypothesis-generating finding. This finding suggests that PCAT_MA_ could be related to vessel vulnerability for atherosclerosis. This hypothesis should be further investigated in pathophysiological and prospective clinical studies.

To analyze all three coronary arteries, an adjusted measurement method was used in our study. Previously, a measurement length of 40 mm was used [16, 21, 25] which was feasible for the RCA because it has fewer side branches and proximal variations than the LAD and LCX. To avoid influence of side branches, measurement length was reduced from 40 to 10 mm in this study. Results from our sub-study demonstrated no differences in PCAT_MA_ between our 10-mm methods and 40-mm method. PCAT_MA_ measurement width around the coronary could potentially affect measurement accuracy. Prior studies measured PCAT_MA_ using approximately 3 mm thickness (or equal to vessel diameter) around the coronary vessels [16, 20, 21, 25]. However, contrast enhancement of the lumen has been found to influence the HU values in the voxels adjacent to the luminal border [32]. To take this into account, we applied a 1-mm gap around the vessel wall. Thus, our study measured PCAT_MA_ using a more constrictive measurement width, making it more suitable for LAD and LCX measurements, and potentially more sensitive to inflammatory changes. Manual PCAT measurements using the method described here can be performed in a similar time span compared with manual EAT measurements. Fully automated software, as mentioned by some researchers [21], allows for PCAT_MA_ evaluation within 30 s, increasing the time efficiency of PCAT_MA_ analysis and enabling use in clinical practice.

While PCAT_MA_ and EAT both are measures of adipose tissue, they represent different processes [16]. PCAT_MA_ quantifies fat at the per-vessel level or per-lesion level as an indicator of coronary inflammation, while EAT provides a measure of the volume of the entire epicardial fat system as a marker for paracrine effects of fat. Thus, PCAT_MA_ may provide a more specific, focal assessment of coronary risk and vulnerability. PCAT_MA_ was shown to have additional diagnostic value with more precision and specificity compared with EAT measurements [33]. Studies found that PCAT_MA_ and FFR were related at the per-vessel level [20] and that PCAT_MA_ of RCA was able to assist in risk stratification of cardiovascular mortality [25]. The combined use of PCAT_MA_, total plaque volume, and diameter stenosis has shown high diagnostic accuracy for prediction of hemodynamically significant coronary stenosis [24]. Interestingly, Goeller et al [21] found that changes in attenuation of adipose tissue in the pericoronary space were related to changes in plaque burden. These results suggest that these effects are associated with changes in PCAT specifically rather than adipose tissue in general.

Besides differences between kV levels and coronary arteries, our results show a slight but important difference in PCAT_MA_ between men and women that could not be explained by differences in kV distribution. Men are known to get CAD more frequently than women and at an earlier age [31]. The PCAT_MA_ difference between men and women follows the same trend. This finding deserves further exploration in a study with comprehensive cardiovascular risk factor assessment and more diverse range of coronary atherosclerosis. Sex-related differences in PCAT_MA_ could be caused by several factors. Men have a higher amount of EAT than women [34, 35], related to cardiovascular risk. There are sex-related differences in the regulation mechanism of pericardial adipokines [36] and in the physiological mechanism of adipose tissue [37]. Additionally, it could be that differences in sex-related hormones, higher low-density lipoprotein in male patients, and differences in risk factors impact PCAT_MA_ in men and women [38].

### Limitations

This was a single-center retrospective study of patients with a clinical indication for cCTA. There was no follow-up, since a normal cCTA result led to discharge from the cardiology outpatient clinic. Some of the patients might have been at higher risk to develop CAD, reflected in already altered PCAT_MA_ values while there was no plaque development yet. There could be factors other than tube voltage affecting PCAT_MA_ measurements such as obesity or diabetes, inflammatory processes, or specific medication. Further work is needed to explore all potentially influencing factors. With regard to the measurement itself, the anatomical variation of LCX compared with LAD and RCA was relatively large and could have affected the PCAT_MA_ measurement. However, with our adjusted measurement protocol, we found excellent correlation between repeated measurements within and between readers with limited bias, indicating the validity of the PCAT_MA_ measurement.

## Conclusion

In conclusion, our results showed that PCAT_MA_ varied considerably by tube voltage in patients without plaque on cCTA, with minor differences in PCAT_MA_ between coronary arteries (LAD, LCX, RCA). cCTA kV setting needs to be taken into account when interpreting PCAT_MA_ values.

## Electronic supplementary material

ESM 1 (DOCX 374 kb)

## Abbreviations

BMI: Body mass index
CAD: Coronary artery disease
cCTA: Coronary computed tomography angiography
EAT: Epicardial adipose tissue
ECG: Electrocardiography
kV: Kilovoltage
LAD: Left anterior descending coronary artery
LCX: Left circumflex coronary artery
PCAT_MA_: Pericoronary adipose tissue mean attenuation
RCA: Right coronary artery
SD: Standard deviation
