# Supplementary material for: Towards reference values of pericoronary adipose tissue attenuation: impact of coronary artery and tube voltage in coronary computed tomography angiography
Source: Eur Radiol. 2020 Jul 22;30(12):6838–46. doi: 10.1007/s00330-020-07069-0 (PMC7599179; doi:10.1007/s00330-020-07069-0)

Table S1. Mean and SD of PCAT_MA_ for different measurement lengths.

| PCAT_MA_ | 40mm Mean (HU) | 40mm SD (HU) | 10mm Mean (HU) | 10mm SD (HU) | p Value |
| --- | --- | --- | --- | --- | --- |
| LAD | -95.6 | 9.7 | -94.5 | 11.0 | 0.124 |
| LCX | -88.7 | 10.0 | -90.0 | 8.7 | 0.118 |
| RCA | -92.9 | 8.3 | -91.6 | 9.7 | 0.116 |

PCAT_MA_ is pericoronary adipose tissue mean attenuation; LAD is left anterior descending coronary artery; LCX is left circumflex coronary artery; RCA is right coronary artery; HU is Hounsfield Units; SD is standard deviation

Table S2. Intra- and inter-observer comparison for PCAT_MA_ measurements.

PCAT_MA_ is pericoronary adipose tissue mean attenuation; HU is Hounsfield Units; SD is standard deviation.

|  | First PCAT_MA_  Reading ± SD (HU) | Second PCAT_MA_  Reading ± SD (HU) | p value comparison readings | Intra-class correlation coefficient | p value  correlation |
| --- | --- | --- | --- | --- | --- |
| Intra-reader comparison | -91.3±9.8HU | -91.6±9.7HU | p=0.255 | 0.982 | p<0.001 |
| Inter-reader  comparison | -91.3±9.8HU | -91.6±9.7HU | p=0.364 | 0.974 | p<0.001 |

Figure S1. Bland-Altman plot for PCAT_MA_ measurement length of 40mm and 10mm in LAD (A), LCX (B) and RCA (C).

PCAT_MA_ is pericoronary adipose tissue mean attenuation; LAD is left anterior descending coronary artery; LCX is left circumflex coronary artery; RCA is right coronary artery; HU is Hounsfield Units.


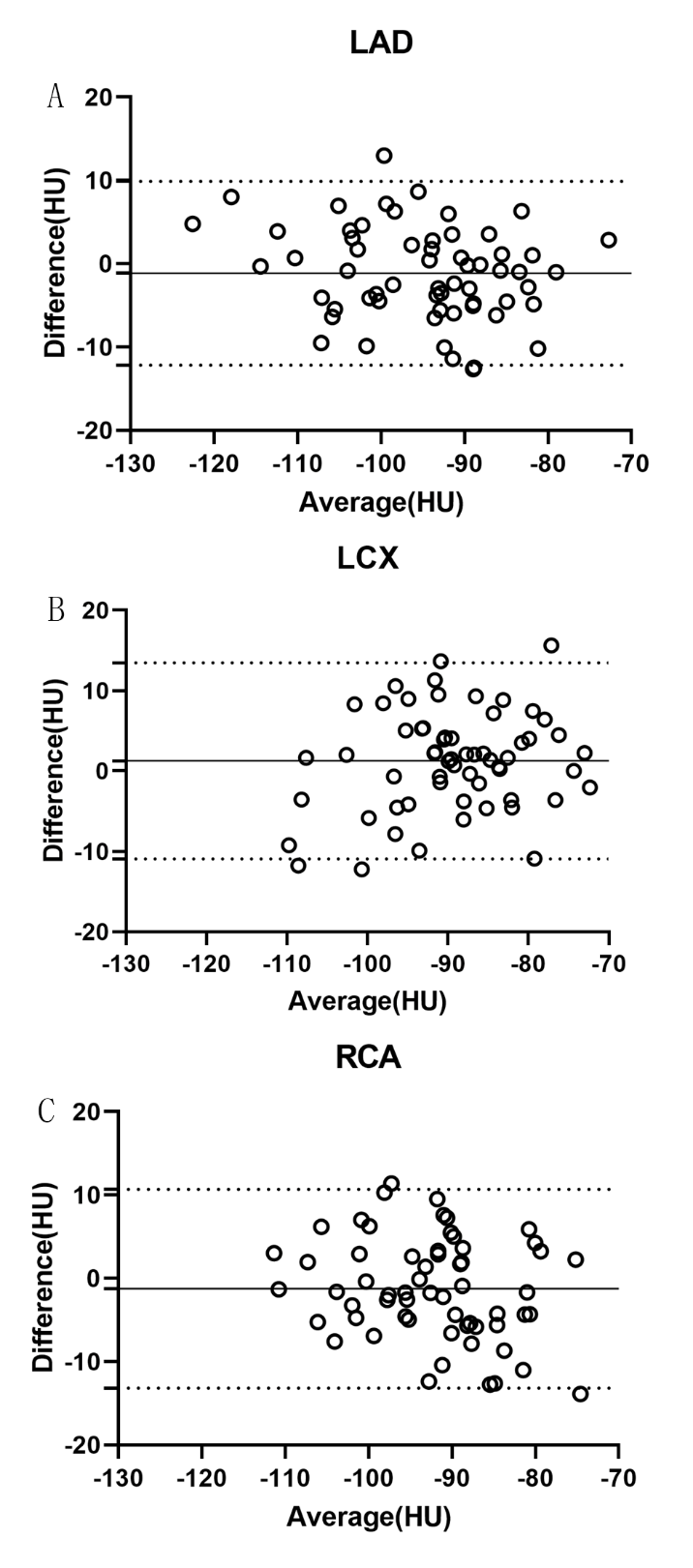


Figure S2. Bland-Altman plot for intra- (A) and inter-reader agreement (B) in PCAT_MA_.

PCAT_MA_ is pericoronary adipose tissue mean attenuation; HU is Hounsfield Units.


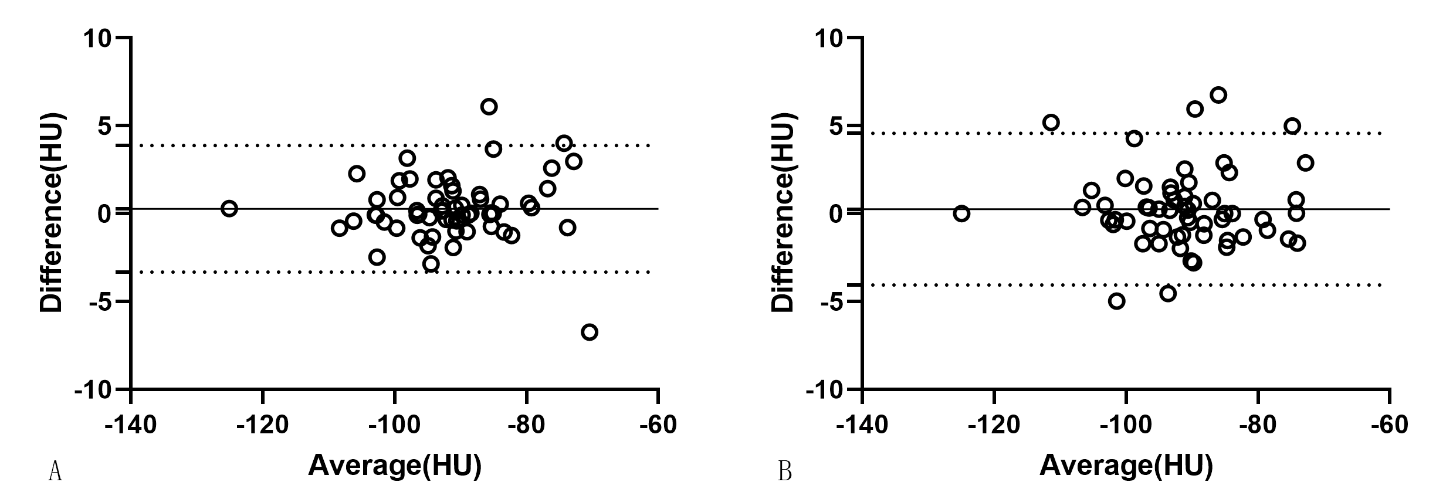

Supplement: Supplementary file 1 — (DOCX 374 kb) [file 330_2020_7069_MOESM1_ESM.docx]
